# Iron deficiency prevalence among pregnant women in Cambodia varies widely by trimester, inflammation adjustments, and across different ferritin thresholds

**DOI:** 10.1371/journal.pgph.0004650

**Published:** 2025-06-04

**Authors:** Stella C. Mlewa, Lulu X. Pei, Cassandra Sauer, Colleen C. Farrell, Hou Kroeun, Mam Borath, Tim J. Green, Kyly C. Whitfield, Crystal D. Karakochuk

**Affiliations:** 1 Food, Nutrition, and Health, The University of British Columbia, Vancouver, British Columbia, Canada; 2 BC Children’s Hospital and Women’s Health Research Institutes, Vancouver, British Columbia, Canada; 3 Helen Keller International, Phnom Penh, Cambodia; 4 National Sub-Committee for Food Fortification, Cambodia Ministry of Planning, Phnom Penh, Cambodia; 5 College of Nursing and Health Sciences, Flinders University, Bedford Park, South Australia, Australia; 6 Applied Human Nutrition, Mount Saint Vincent University, Halifax, Nova Scotia, Canada; Yale University, UNITED STATES OF AMERICA

## Abstract

Iron deficiency (ID) prevalence has been consistently reported as low among non-pregnant women in Cambodia, but less is known about iron status during pregnancy. Assessing iron status during pregnancy is critical, as deficiency can increase the risk of adverse pregnancy outcomes. We assessed anemia, ID, and inflammation prevalence in a cohort of pregnant women in Cambodia. Venous blood from 90 pregnant women (12–32 weeks’ gestation) was collected before the start of a 2016 trial conducted in Prey Veng province. Gestational age was recorded on the same day as blood collection. Hemoglobin was measured on a hematology autoanalyzer, and ferritin, α-1 acid glycoprotein (AGP), and C‐reactive protein (CRP) concentrations were measured with a sandwich-ELISA. Ferritin concentrations are presented as unadjusted and inflammation-adjusted (based on AGP and CRP concentrations). ANOVA and post-hoc pairwise t-tests were used to compare variables across trimesters of pregnancy. Mean±SD age of women was 26 ± 5 years. Most women (94%) reported consumption of iron and folic acid (IFA) tablets during pregnancy (mean±SD: 85 ± 19 tablets), and 72% received deworming treatment. Overall, 49% of women had anemia (hemoglobin <110 g/L for first and third trimesters; < 105 g/L for second trimester); with 43%, 34%, and 64% in the first, second and third trimester, respectively. ID prevalence (unadjusted ferritin <30 µg/L) ranged widely by trimester: 0%, 17% and 76% in the first, second and third trimester, as well as with use of a lower ferritin threshold (0–52%; < 15 µg/L), and whether ferritin was inflammation-adjusted (61% with and 43% without adjustment; < 30 µg/L). ID prevalence was high among women in third trimester, despite high IFA compliance. These findings underscore the need to consider the trimester of pregnancy in anemia and ID assessment. More research is needed to determine if trimester-specific thresholds for ferritin in pregnant populations are warranted and whether ferritin should be adjusted for inflammation in pregnancy.

## Introduction

Accurate assessment of iron status during pregnancy is crucial as deficiency can increase the risk of adverse maternal and fetal outcomes, such as postpartum hemorrhage, low birthweight or small for gestational age infants, and stillbirth [1,2]. Prenatal iron deficiency (ID) is one of the leading causes of anemia in infants and young children [3]. Iron requirements during pregnancy are increased to support maternal red blood cell mass expansion and the growing fetus and placenta [4].

The Cambodian Ministry of Health, in alignment with the global World Health Organization (WHO) policy, recommends the consumption of daily oral iron and folic acid (IFA) supplements during pregnancy (30–60 mg iron and 0.4 mg folic acid) to address these increased iron requirements and prevent and reduce ID during pregnancy [5,6]. Recently, there has been a global push to replace IFA with multiple micronutrient supplements (MMS; which includes 30 mg iron, 0.4 mg folic acid, and 13 other vitamins and minerals), based on evidence demonstrating its superiority over IFA on positive birth outcomes [7,8]. In a review conducted in 2019, Keats et al. concluded that supplementation with MMS during pregnancy reduced the risk of low birth weight by 12% and the risk of small for gestational age by 8%, compared with IFA [9]. Based on this review and other critical evidence [10], the WHO updated its antenatal care guidelines in 2020 to recommend MMS when ‘rigorous research’ (e.g., acceptability trials) justified its implementation [11]. The Cambodian Ministry of Health has expressed strong interest in transitioning from using IFA to MMS during pregnancy and has commenced implementation research to inform this important policy change [12,13].

What is left to ascertain, in light of this potential policy change, is whether the lower dose of iron in MMS is sufficient to prevent ID in pregnancy in Cambodian women. The prevalence of ID has been consistently reported as low among non-pregnant women in Cambodia; however, less is known about the iron status of pregnant women. It is critical to understand the scope of this matter in order to inform policy and program guidance for the delivery of MMS.

Given the scarcity of data on ID prevalence in pregnant women and the timeliness of the potential national policy change to recommend MMS during pregnancy in Cambodia, we assessed the prevalence of anemia, ID, and inflammation in a cohort of pregnant women using blood specimens collected in a previously-conducted trial in Cambodia.

## Materials and methods

### Study design

We obtained baseline blood specimens from a clinical trial conducted in Prey Veng province, Cambodia [14]. In this trial, 90 pregnant women between 12- and 32-weeks’ gestation were enrolled in a double-blind randomized clinical trial, which was conducted primarily to determine if consumption of thiamine-fortified fish sauce resulted in higher erythrocyte thiamine diphosphate concentrations among lactating women and newborn infants as compared with a control sauce [14]. The recruitment period for this trial was between September 15^th^ and October 9^th^, 2014, and results have been published [14]. Ethical approval for the study was granted by the Cambodian National Ethics Committee for Health Research (0245-HECHR), The University of British Columbia (UBC) Clinical Research Ethics Board (H14-00103) and the UBC BC Children’s and Women’s Research Ethics Board (H14-01654). The trial was registered at clinicaltrials.gov (NCT02221063). Women provided written informed consent to participate. Additional information regarding the ethical, cultural, and scientific considerations specific to inclusivity in global research is included in the Supporting Information (S1 Checklist).

### Recruitment and eligibility

Women were recruited to the original trial based on the following eligibility criteria: aged 18–45 years and between 12- to 32-weeks’ pregnant with a singleton fetus (self-reported), female head of their household, planning to exclusively breastfeed their infant for 6 months, no history of pre-eclampsia, preterm delivery, or birth defects, not involved in other non-governmental nutrition programs, not consuming thiamine-containing dietary supplements, agreement to exclusively consume the fish sauce provided by the study in their household, and no plans to leave their village for the duration of the study period [14].

### Social, demographic and health data collection

Demographic information was collected using an interviewer-administered questionnaire in the women’s homes. Gestational age was self-reported based on the last menstrual period, with assistance from the participants’ antenatal health care team. Women’s gestational age was recorded on the same day as blood specimen collection.

### Blood collection, processing and assessment

Non-fasting venous blood specimens were collected into evacuated EDTA–coated tubes (Becton Dickinson, Franklin Lakes, USA) from women at a central village location. Blood specimens were transported on ice to the National Institute of Public Health Laboratory (NIPHL) in Phnom Penh within 5 hours of collection. Blood was centrifuged at 3000 rpm for 15 minutes at 4°C, and plasma was removed and frozen at -80°C. Hemoglobin (g/L) was measured using an automated hematology analyzer (Sysmex XT-1800i; Sysmex Corporation, Kobe, Japan) at NIPHL in Phnom Penh. Plasma was analyzed for ferritin concentration (µg/L), soluble transferrin receptor (sTfR, mg/L), α-1 acid glycoprotein (AGP, g/L), and C‐reactive protein (CRP, mg/L), with a sandwich-ELISA (VitMin Lab, Willstaett, Germany) [15].

### Statistical analysis

Demographic and health characteristics are summarized as proportions (*n*, %), mean ± standard deviation (SD) values for normally distributed variables, and median and interquartile ranges (IQR) for variables with skewed distributions. Using AGP and CRP12 biomarkers, ferritin and sTfR concentrations were adjusted for inflammation following Biomarkers Reflecting Inflammation and Nutritional Determinants of Anemia (BRINDA) guidelines [16,17]; both unadjusted and adjusted values are presented. Hemoglobin and unadjusted ferritin concentrations were compared across trimesters using ANOVA and post-hoc pairwise t-tests (or Kruskal-Wallis and post-hoc pairwise Dunn’s tests for skewed distributions). WHO trimester-specific thresholds for hemoglobin were used to define anemia during pregnancy: < 110 g/L for the first and third trimesters and <105 g/L for the second trimester [18]. Lastly, we assessed ID using two thresholds of ferritin concentration <15 µg/L [19] and <30 µg/L [20], as well as considered the physiologically based trimester-specific thresholds for ferritin that were recently proposed by Mei et al. [21]: unadjusted ferritin <25 µg/L for the first trimester and <20 µg/L for the second and third trimesters. All analyses were performed in Stata statistical software (StataCorp LLC, College Station, USA).

## Results

### Demographic and health characteristics

Ninety pregnant women from Prey Veng who met the study’s trial eligibility criteria were enrolled. Mean age, parity, and the proportion of study participants who consumed IFA and deworming tablets are reported in Table 1. Most women reported consuming IFA and deworming tablets during pregnancy.

**Table 1:** Demographic and health data of enrolled pregnant women in Cambodia^1^.

Age, years	26.3 ± 5.0
Gestational age^2^, weeks	24.4 ± 7.0
Trimester of pregnancy at enrollment^2^	
First (0–12 weeks’ gestation)	7/90 (8%)
Second (13–27 weeks’ gestation)	41/90 (46%)
Third (28 + weeks’ gestation)	42/90 (47%)
Parity^3^	
0 children	43/87 (49%)
1-2 children	41/87 (47%)
3-4 children	3/87 (4%)
Women who consumed IFA supplements in the current pregnancy^3^	82/87 (94%)
Number of IFA tablets consumed in the current pregnancy^3^	84.6 ± 18.5
Deworming tablet consumed in the current pregnancy^3^	63/87 (72%)

^1^Total *n* = 90. Values are mean±SD or *n* (%). IFA, iron and folic acid; SD, standard deviation.

^2^Based on self-reported gestational age at the time of baseline blood specimen collection.

^3^Based on self-reported data collected after delivery; *n* = 3/90 values missing due to loss to follow up after delivery.

### Hematologic indicators and biomarkers of iron deficiency and inflammation

The prevalence of anemia, micronutrient deficiencies, and acute and chronic inflammation at baseline (before the trial commenced) among all enrolled women, and by trimester of pregnancy, is summarized in Table 2. The prevalence of anemia and ID ranged widely from first to third trimester and was largely influenced by inflammation-adjustment (for ferritin and sTfR) and by the threshold applied for hemoglobin and ferritin (for anemia and ID diagnosis, respectively). The prevalence of acute inflammation was very low and there was no biochemical evidence of chronic inflammation.

**Table 2:** Prevalence of anemia, iron deficiency, and inflammation among all enrolled pregnant women and by trimester of pregnancy^1^.

	All pregnant women	First trimester	Second trimester	Third trimester
Total, *n* (%)	90/90 (100%)	7/90 (8%)	41/90 (46%)	42/90 (47%)
**Anemia**				
Hemoglobin concentration, g/L	107.7 ± 10.2	112.9 ± 11.8	109.1 ± 10.3	105.5 ± 9.5
Anemia prevalence, based on WHO trimester-specific Hb thresholds^2^	44/90 (49%)	3/7 (43%)	14/41 (34%)	27/42 (64%)
**Iron deficiency**				
Ferritin concentration, unadjusted, µg/L	35.5 (16.4, 66.5)	114.4 (57.3, 148.1)	50.4 (33.0, 82.8)	14.0 (8.6, 25.9)
WHO recommendation for use of unadjusted ferritin <15 µg/L [19]	22/90 (24%)	0/7 (0%)	0/41 (0%)^3^	22/42 (52%)^3^
ACOG recommendation for use of unadjusted ferritin <30 µg/L [20]	39/90 (43%)	0/7 (0%)	7/41 (17%)	32/42 (76%)
Recently proposed physiologically based trimester-specific thresholds: unadjusted ferritin <25 µg/L for first trimester and <20 µg/L for second and third trimesters [21]	25/90 (28%)	0/7 (0%)	0/41 (0%)	25/42 (59%)
Ferritin concentration, inflammation-adjusted^4^, µg/L	24.8 (10.7, 39.3)	43.1 (35.8, 149.0)	32.1 (24.4, 42.4)	9.4 (6.5, 19.0)
Inflammation-adjusted ferritin^4^ < 15 µg/L	32/90 (36%)	0/7 (0%)	3/41 (7%)	29/42 (69%)
Inflammation-adjusted ferritin^4^ < 30 µg/L	55/90 (61%)	1/7 (14%)	19/41 (46%)	35/42 (83%)
STfR concentration, unadjusted, mg/L	4.1 (3.1, 5.7)	3.6 (2.2, 4.7)	3.6 (2.8, 4.1)	5.2 (4.1, 6.5)
Unadjusted sTfR > 8.3 mg/L	4/90 (4%)	0/7 (0%)	0/41 (0%)	4/42 (10%)
STfR concentration, inflammation-adjusted^4^, mg/L	3.3 (2.5, 4.6)	2.3 (1.6, 3.3)	2.7 (2.2, 3.2)	4.3 (3.5, 5.2)
Inflammation-adjusted sTfR^4^, > 8.3 mg/L	2/90 (2%)	0/7 (0%)	0/41 (0%)	2/42 (5%)
**IDA**				
WHO trimester-specific Hb thresholds^2^ and unadjusted ferritin <15 µg/L	16/90 (18%)	0/7 (0%)	0/41 (0%)	16/42 (38%)
WHO trimester-specific Hb thresholds^2^ and unadjusted sTfR > 8.3 mg/L	3/90 (3%)	0/7 (0%)	0/41 (0%)	3/42 (7%)
**Inflammation**				
CRP, mg/L	1.0 (0.6, 1.8)	1.6 (0.4, 1.8)	0.9 (0.4, 1.6)	1.1 (0.8, 2.0)
Acute inflammation, CRP > 5 mg/L	10/90 (11%)	0/7 (0%)	5/41 (12%)	5/42 (12%)
AGP, g/L	0.4 (0.3, 0.5)	0.4 (0.3, 0.6)	0.4 (0.4, 0.5)	0.4 (0.3, 0.5)
Chronic inflammation, AGP > 1 mg/L	0/90 (0%)	0/7 (0%)	0/41 (0%)	0/42 (0%)

^1^*n* = 90. Values are mean±SD for normally distributed variables, median (IQR) for variables with skewed distributions, or *n* (%). ACOG, American College of Obstetrics and Gynecology; AGP, α-1 acid glycoprotein; CRP, C-reactive protein; Hb, hemoglobin; IDA, iron deficiency anemia; IQR, interquartile range; SD, standard deviation; sTfR, soluble transferrin receptor; WHO, World Health Organization.

^2^WHO trimester-specific thresholds for hemoglobin: < 110 g/L for first and third trimesters; < 105 g/L for the second trimester [18].

^3^WHO only recommends use of this threshold for the first trimester of pregnancy [19]; thus, prevalence rates in second and third trimesters are presented here for demonstrate what prevalence would be if the same ferritin threshold was applied.

^4^Ferritin and sTfR values were adjusted for inflammation based on CRP and AGP concentrations as per BRINDA guidelines [16,17].

**Fig 1:**
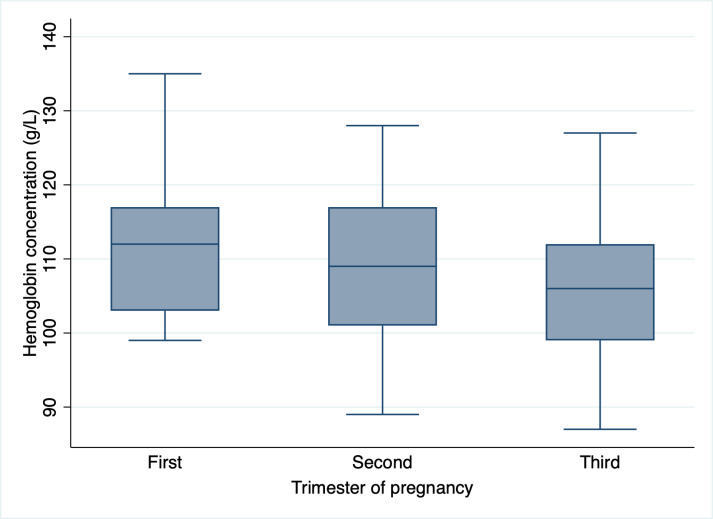
Hemoglobin concentration (g/L), by trimester of pregnancy. Trimester: First (*n* = 7), Second (*n* = 41), Third (*n* = 42).

**Fig 2:**
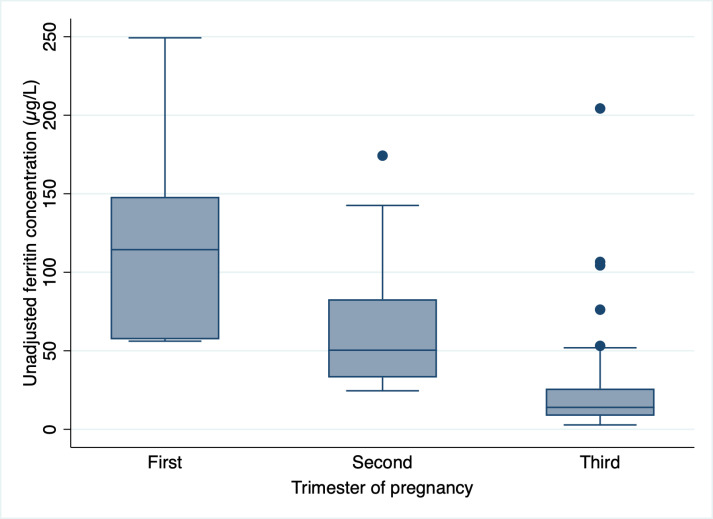
Unadjusted ferritin concentration (µg/L), by trimester of pregnancy. Trimester: First (*n* = 7), Second (*n* = 41), Third (*n* = 42).

Hemoglobin and unadjusted ferritin concentrations in women by trimester of pregnancy are presented in Figs 1 and 2, respectively. Both biomarkers were observed to decrease from the first to third trimester of pregnancy. In assessing trends for hemoglobin concentration, we found that values did not significantly differ from the first to third trimester (*p* = 0.076). However, for unadjusted ferritin concentrations, there was a significant decrease in values from the first to third trimester (*p* < 0.001).

## Discussion

Our objective of this study was to assess ID prevalence among a cohort of pregnant individuals in Cambodia, in light of the recent policy consideration to transition from recommending IFA (including 60 mg iron) during pregnancy to MMS (including only 30 mg iron), and the lack of current evidence on ID prevalence in pregnant individuals in Cambodia.

We found that ID prevalence (unadjusted ferritin <30 µg/L) ranged widely by trimester: 0%, 17% and 76% in the first, second and third trimester, respectively. ID prevalence also ranged widely across trimesters with use of a lower ferritin threshold (0–52%; unadjusted ferritin <15 µg/L), and whether ferritin was inflammation-adjusted (61% with and 43% without adjustment; < 30 µg/L). Regardless of these variations, ID prevalence in the second and third trimesters was higher than we expected (~17% in second trimester and ~76% in third trimester based on unadjusted ferritin <30 µg/L), given that much of the previous research among non-pregnant women of reproductive age in Cambodia has showed very low ID prevalence (ranging from 3-13% based on ferritin <15 µg/L) [22–24]. It is well established that in the second and third trimesters, demands for iron increase as a result of the growing demands of the fetus (and placenta) and considerable blood volume expansion [25]. However, these ID rates in later gestation are higher than expected, especially as nearly all women in our study reported high compliance to IFA supplementation and deworming tablets during the pregnancy. Pregnant women in our study reported to have consumed IFA for ~84 days, which is in line with the current Cambodian national guidelines which recommend IFA for 90 days [6].

In 2024, the WHO updated their recommendations for anemia assessment in pregnancy, with the revision of the trimester-specific hemoglobin thresholds for anemia diagnosis: < 110 g/L for the first and third trimesters, and <105 g/L in the second trimester [18]. Of note, these trimester-specific thresholds were established based on statistical modelling (thresholds for each trimester represent the 5^th^ percentile for hemoglobin concentration in a globally diverse population of apparently healthy individuals) [18]. The different thresholds across trimesters are thought to reflect the changing physiological processes during pregnancy, including second trimester blood volume expansion, and third trimester changes in inflammatory measures [26]. These thresholds, however, have yet to be linked to clinical outcomes of importance such as pregnancy and birth characteristics. Despite these shortcomings in how these thresholds were developed, there is a justification for the trimester-specific thresholds for hemoglobin.

Conversely to hemoglobin, trimester-specific thresholds have not yet been proposed for ferritin (despite the more pronounced and significant drop in biomarker concentrations across the three trimesters – as observed in Fig 2). Thus, there remains a clear gap both in the evidence and in the recommendations for ID assessment in pregnancy. The 2020 WHO guideline on the use of ferritin concentrations to assess iron status recommends that ferritin (<15 µg/L) should only be used for ID assessment in the first trimester of pregnancy. Caution is raised for the use of ferritin to assess ID in the second and third trimesters due to the similar issues noted above for hemoglobin (the changing physiological processes during pregnancy, particularly in second and third trimesters). Thus, no thresholds are specifically outlined for the second and third trimesters. In an American practice bulletin (updated in 2021), the ACOG suggests that a serum ferritin >30 µg/L during any trimester of pregnancy is considered as ‘normal’ [20]. And more recently, Mei et al. proposed physiologically based trimester-specific serum ferritin thresholds for ID (~25 µg/L in the first and ~20 µg/L in the second and third trimesters). These values were estimated based on statistical analyses which tracked hepcidin (the iron-regulating peptide hormone) concentrations in a United States cohort using previously collected data from the National Health and Nutrition Examination Survey (NHANES). Given that there are clear discrepancies across these guidelines and practice bulletins on which ferritin threshold is optimal during pregnancy, more research is needed to evaluate trimester-specific thresholds for ID in pregnant populations.

Further, it remains unclear whether ferritin concentrations should be adjusted for inflammation in pregnant populations. The Biomarkers Reflecting Inflammation and Nutritional Determinants of Anemia (BRINDA) project has released globally endorsed guidelines that recommend ferritin and sTfR concentrations be adjusted for inflammation (based on AGP and CRP concentrations) in preschool children and non-pregnant women of reproductive age [16,17]; however, it remains uncertain whether these biomarkers should be adjusted in pregnant populations. It is also unclear if this investigation has yet to be undertaken, or if for reasons not indicated, inflammation adjustment in pregnant populations is not warranted. Ultimately, more research is needed to determine whether values should be adjusted for inflammation in pregnancy, as the decision to adjust or not to adjust can have a substantial influence on ID prevalence rates, as observed in our current study.

As a result of the lack of consensus around these issues, we are left with some uncertainty in our estimates of ID prevalence during pregnancy in our Cambodian cohort. However, what is certain is that our findings underscore the need to consider the trimester of pregnancy in anemia and ID assessment. Data on gestational age should be collected at the time of blood specimen collection, to accurately estimate trimester of pregnancy and the appropriate trimester-specific threshold to apply for hemoglobin (and potentially ferritin).

The Ministry of Health in Cambodia has aligned its efforts with the Sustainable Development Goals (SDGs), in order to optimize maternal and infant health and promote wellbeing, which includes the goal to reduce and prevent iron deficiency during pregnancy. Currently, the Ministry of Health in Cambodia is considering transitioning from the use of IFA to MMS during pregnancy, thus, confirming the diagnostic approaches to screen and treat ID during pregnancy is critical. Assessment of whether MMS (including 30 mg iron) is as effective as IFA (including 60 mg iron) in preventing ID in pregnant women in Cambodia is also warranted to inform new policy and optimize pregnancy outcomes and the health of mothers and infants in Cambodia.

A strength of this study is that we provide estimates of ID prevalence among pregnant women using a variety of approaches and guidelines, in light of the scarcity of data in this population. We present unadjusted and inflammation-adjusted ferritin and sTfR concentrations using globally endorsed BRINDA methods; however, we recognize there is limited evidence for the use of BRINDA adjustments in pregnant populations. Lastly, we acknowledge our small sample size, that our study population was limited to only one province, and that data on smoking status was not collected to ascertain if hemoglobin concentrations should be adjusted for this exposure [18]; however, evidence from the nationally representative Demographic and Health Survey in 2021–22 suggests that tobacco smoking (daily or even occasional use of any tobacco products) among women 15–49 years is not a common practice (<1% prevalence in Prey Veng province) [27].

In conclusion, ID prevalence was surprisingly high among pregnant women in the second and third trimesters of pregnancy, despite high compliance to daily IFA supplementation. This may be due, in part, to the normal physiological changes during pregnancy, such as blood volume expansion and hemodilution (duly acknowledging the current lack of consensus around trimester-specific ferritin thresholds during pregnancy). It also may be attributed to the low bioavailability of the form of supplemental iron used in IFA tablets (i.e., ferrous iron salts), or due to the presence of iron absorption inhibitors in the Cambodian diet (e.g., phytates), which may contribute to inadequate iron absorption. However, we are ultimately left with some uncertainty in our estimates of ID prevalence during pregnancy in Cambodian women due to conflicting ferritin thresholds in ID assessment using current available guidelines and uncertainty whether to adjust for the effect of inflammation in pregnant populations.

## Supporting information

S1 Checklist Inclusivity in global research. (DOCX)

S1 Data Original data file. (DTA)
